# Properties of the transient outward, ultra-rapid delayed rectifier and acetylcholine-sensitive potassium currents in isolated atrial myocytes from dogs: sinus rhythm and tachypaced model of permanent atrial fibrillation

**DOI:** 10.1186/1471-2210-11-S2-A60

**Published:** 2011-09-05

**Authors:** Zsófia Kohajda, Attila Kristóf, Péter P Kovács, Claudia Corici, László Virág, Viktor Juhász, Zoltán Husti, István Baczkó, András Varró, Norbert Jost

**Affiliations:** 1 Department of Pharmacology and Pharmacotherapy, University of Szeged; Division of Cardiovascular Pharmacology, Hungarian Academy of Sciences, 6720 Szeged, Hungary

## Background

Atrial fibrillation (AF) is a common and severe arrhythmia, which largely affects quality of life. State-of-the-art treatment of AF still relies heavily on pharmacological modalities. Therefore, the aim of the present study was to investigate and compare the properties of three repolarizing currents which contribute to AF-induced remodeling, i.e. the transient outward (I_to_), ultra-rapid delayed rectifier (I_Kur_) and acetylcholine-sensitive potassium currents (I_K,ACh_) in isolated atrial myocytes obtained from normal (SR) and tachypaced model of permanent atrial fibrillation (ATR) dogs.

## Methods

The tachypaced atrial fibrillation model was performed in dogs. Transmembrane ionic currents were investigated by applying the whole-cell patch clamp technique at 37°C, and ECG was recorded in conscious dogs.

## Results

In all atrial canine myocytes, we have identified an I_to_ current sensitive to 4-aminopyridine (4-AP; 3 mM). The current inactivation was best fitted by two exponentials. The I_to_ current was slightly downregulated in ATR cells when compared with that recorded in SR cells. The I_Kur_ current, measured as sustained current (I_sus_), was upregulated in ATR dogs. However, the selective I_Kur_ blocker 4-AP (50 µM) did not block either I_sus_ or I_Kur_ „like tail” currents, which questions the reliability of these results. I_K,ACh_ was activated by the cholinergic agonist carbachol (CCh; 2 µM). In SR, CCh activated a large current either at inward or outward directions. The selective I_K,ACh_ blocker tertiapin (10 nM) blocked the CCh-induced current by 57%. In atrial myocytes from ATR dogs we could measure the presence of a constitutively active I_K,ACh_, which could be blocked by 26 % with 10 nM tertiapin. However, in ATR atrial myocytes, CCh in addition could also activate a significant ligand-dependent and tertiapine-sensitive I_K,ACh_ current. Tertiapin effectively prevented burst-induced AF in conscious ATR dogs.

## Conclusions

The presence of the constitutively activated I_K,ACh_ in atrial myocytes from ATR dogs shows that electrical remodeling developed in our model; this was further supported by the inducibility of AF by rapid atrial bursts in these dogs. The I_K,ACh_ current (both ligand-dependent and constitutively active currents) seems to play a significant role in the canine atrial electrical remodelling, and may be a promising drug target for suppressing AF.

